# Wayfinding artificial intelligence to detect clinically meaningful spots of retinal diseases: Artificial intelligence to help retina specialists in real world practice

**DOI:** 10.1371/journal.pone.0283214

**Published:** 2023-03-27

**Authors:** Hideki Shiihara, Shozo Sonoda, Hiroto Terasaki, Kazuki Fujiwara, Ryoh Funatsu, Yousuke Shiba, Yoshiki Kumagai, Naoto Honda, Taiji Sakamoto

**Affiliations:** 1 Department of Ophthalmology, Kagoshima University Graduate School of Medical and Dental Sciences, Kagoshima, Japan; 2 Sonoda Eye Clinic, Kagoshima, Japan; 3 NIDEK CO., LTD., Gamagori, Japan; University of Miami Hospital, UNITED STATES

## Abstract

**Aim/background:**

To aim of this study is to develop an artificial intelligence (AI) that aids in the thought process by providing retinal clinicians with clinically meaningful or abnormal findings rather than just a final diagnosis, i.e., a “wayfinding AI.”

**Methods:**

Spectral domain optical coherence tomography B-scan images were classified into 189 normal and 111 diseased eyes. These were automatically segmented using a deep-learning based boundary-layer detection model. During segmentation, the AI model calculates the probability of the boundary surface of the layer for each A-scan. If this probability distribution is not biased toward a single point, layer detection is defined as ambiguous. This ambiguity was calculated using entropy, and a value referred to as the ambiguity index was calculated for each OCT image. The ability of the ambiguity index to classify normal and diseased images and the presence or absence of abnormalities in each layer of the retina were evaluated based on the area under the curve (AUC). A heatmap, i.e., an ambiguity-map, of each layer, that changes the color according to the ambiguity index value, was also created.

**Results:**

The ambiguity index of the overall retina of the normal and disease-affected images (mean ± SD) were 1.76 ± 0.10 and 2.06 ± 0.22, respectively, with a significant difference (p < 0.05). The AUC used to distinguish normal and disease-affected images using the ambiguity index was 0.93, and was 0.588 for the internal limiting membrane boundary, 0.902 for the nerve fiber layer/ganglion cell layer boundary, 0.920 for the inner plexiform layer/inner nuclear layer boundary, 0.882 for the outer plexiform layer/outer nuclear layer boundary, 0.926 for the ellipsoid zone line, and 0.866 for the retinal pigment epithelium/Bruch’s membrane boundary. Three representative cases reveal the usefulness of an ambiguity map.

**Conclusions:**

The present AI algorithm can pinpoint abnormal retinal lesions in OCT images, and its localization is known at a glance when using an ambiguity map. This will help diagnose the processes of clinicians as a wayfinding tool.

## Introduction

The advent of deep learning has spurred remarkable developments in artificial intelligence (AI) [1]. AI has also been applied in the classification of various images in the field of ophthalmology, with reports emphasizing the possibility of diagnosis based on images with high accuracy. Some studies have even begun working on clinical AI applications [2–11]. However, a major drawback of AI is its inability to show specific clinical findings based on AI diagnosis from images; this is referred to as black box AI [12]. To date, heatmaps have been added to help understand the region of the fundus image referenced to some extent [13, 14]. However, this only complements AI diagnosis.

Recently, Adler-Milstein et al. proposed the concept of Wayfinding AI for next-generation diagnostics [15]. “Wayfinding” is defined as interpreting the context and providing cues that guide a diagnostician. More specifically, AI immediately presents a final diagnosis. However, for medical professionals and patients, the diagnostic journey begins by removing uncertain information from large numbers of data. Typically, a care plan is developed during this journey. A final diagnosis from the outset can never garner the trust of clinicians, emphasizing the need for wayfinding AI, a next-generation AI system that advises on the process, as opposed to the current AI, which handles the final diagnoses.

Optical coherence tomography (OCT) is an instrument for irradiating tomographic images of tissues based on the interferometric property of light and is widely used in numerous medical fields. Particularly in the field of ophthalmology, it is an indispensable tool for the diagnosis of retinal and glaucoma diseases and is widely used globally [16]. In 2019, Seeboeck et al. reported an AI algorithm for detecting abnormalities in OCT-B scan images of the retina [17]. Although this method is excellent for screening a large number of OCT images, it is difficult to use in real-world clinical practice because it is impractical to examine every section of OCT images of the fundus of every patient with AI at busy retinal clinics. Furthermore, the AI’s diagnosis is so called “black and white,” and it is not clear how much trust should be placed in the points that are considered abnormal. This does not necessarily make it suitable for retinal specialists to use AI in daily clinical practice.

This study aimed to create a useful AI model for retinal specialists in daily practice. In the diagnosis of ocular OCT, cases that confused humans were found to be equally confusing with AI to the same degree [18]. Most importantly, the greatest benefit of AI is its ability to quantify the level of confidence. By using this function, it is possible to numerically express the strength of the confidence of the diagnosis in each spot. By looking at a heat map of these data on the 2D fundus image, a physician can see the spot of structural abnormality/ambiguity at a glance. Subsequently, a physician can re-examine the indicated area carefully using OCT and other devices. Repeating these processes would be a real thinking process that leads to easy diagnosis correction. This AI-based work is a process called wayfinding, in which AI assists the thinking process to reach the correct diagnosis with a retina specialist.

## Methods

This study was approved by the Ethics Committee of Kagoshima University Hospital (Kagoshima, Japan) and was registered with the University Hospital Medical Network (UMIN)Clinical Trials Registry (CTR). The registration title is “UMIN000031747, Research on retinal/choroidal structure analysis by novel image analysis technique and machine learning.” on March 2018. The detailed protocol is available at https://upload.umin.ac.jp/cgi-open-bin/ctr/ctr_view.cgi?recptno=R000036250. Written informed consent was obtained from all participants after an explanation of the procedures to be used and possible complications. All the procedures conformed to the tenets of the Declaration of Helsinki.

The subjects underwent OCT at Sonoda Eye Clinic, Kagoshima, Japan, from January 15, 2019, to March 29, 2019, with the data constituting OCT images of 679 eyes. OCT images of the macular region passing through the foveal centralis were analyzed. Images of poor quality due to opacity of the optic media, poor fixation, etc., as well as images that were inverted due to posterior staphyloma were excluded. From 617 eyes that survived the exclusion criteria, 300 were randomly selected for the analysis.

### Imaging protocol

Imaging was performed using SD-OCT (RS-3000 Advance2) manufactured by NIDEK (Tokyo, Japan). Optical coherence tomography (OCT) was performed horizontally through the fovea centralis. As the OCT data, a 9-mm scan width, 1024 A-scan images, and 50 additive sheets were used. The image was extracted as a 1024 × 512 pixel Microsoft Windows bitmap image (bmp).

### Segmentation AI

**Fig 1:**
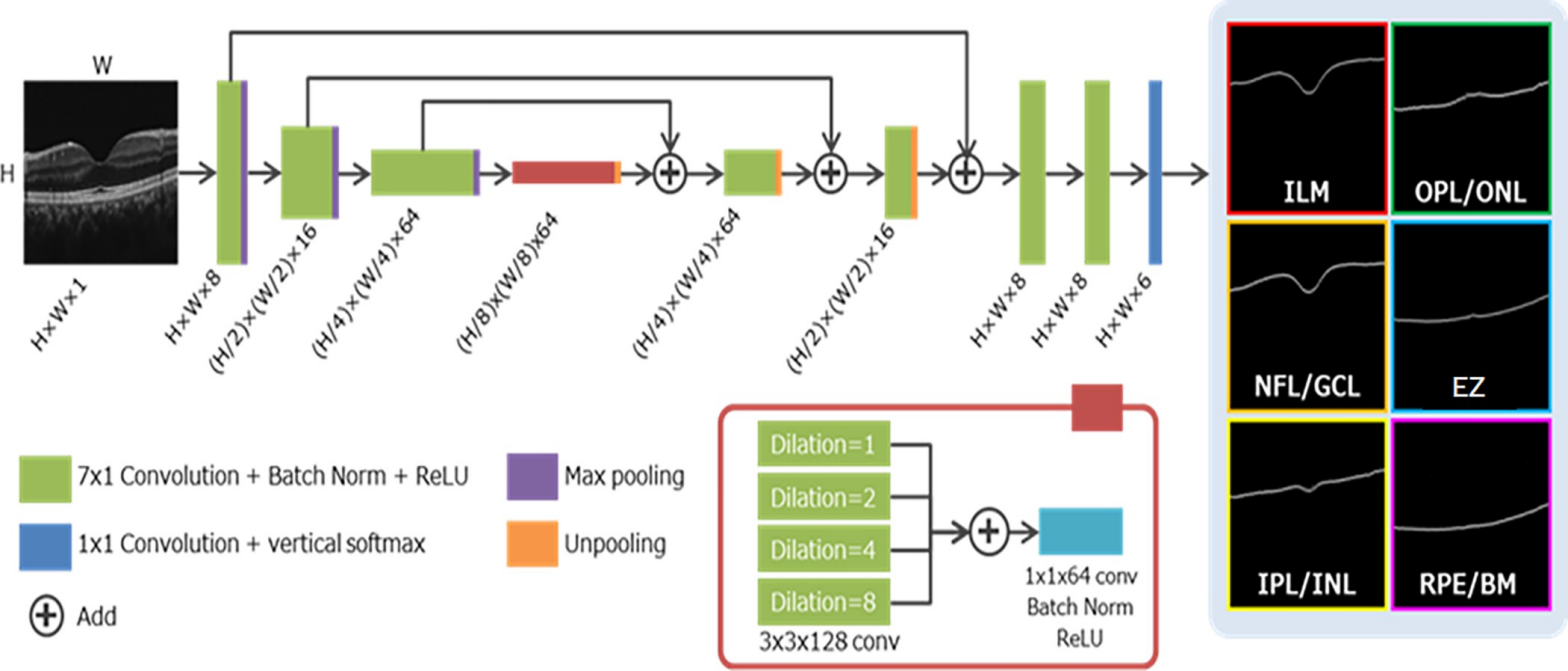
An OCT B-scan automatic image segmentation model was created based on U-Net. The output of this model is the probability distribution of each boundary layer, thus enabling the automatic detection of the boundary for each layer of the retina.

All OCT images were segmented using an OCT B-scan automatic image segmentation model utilizing deep learning [19]. For a brief overview, the segmentation model is based on U-Net [20] and consists of an encoder and decoder, a skip connection between the two, and a multiple dilated convolution (MDC) block. The training of the model was based on previous reports [21]. Briefly, layer boundaries were manually annotated from 171 OCT B-scan images and used as training data. Training was conducted using the AMSGrad optimizer (http://www.Satyenkale.Com/papers/amsgrad.Pdf) using cross-entropy loss function. The batch size was set to 5. To increase the number of training data, the annotated b-scan image was divided vertically into 4 segments. For each segmented image, following data augmentation was applied: left-to-right flipping, scaling (0.9 to 1.1x), and ±15° rotation. Implementation was performed using Sony’s NNabla (https://nnabla.org/). The input was an OCT image, and the output was a probability map for each boundary layer (Fig 1). The encoder and decoder performed a 7×1 vertical convolution to extract the features of the horizontal edges (vertical brightness changes). The MDC block expanded the receptive field by combining convolutions with different dilations to capture the positional relationships of a wide range of features. In the output layer of the model, SoftMax was applied in the vertical direction (the direction of the A-scan). This makes it possible to obtain the probability distribution of the position (depth) of each boundary layer from the A-scan. Finally, the position (depth) with the maximum probability distribution in each A-scan was detected as the boundary layer. This model enables the segmentation of the internal limiting membrane (ILM), nerve fiber layer-ganglion cell layer (NFL-GCL), inner plexiform layer, inner nuclear layer (IPL-INL), outer plexiform layer-outer nuclear layer (OPL-ONL), ellipsoid zone (EZ), and retinal pigment epithelium-Bruch’s membrane (RPE-BM) boundaries (S1 Fig).

### Calculating degree of ambiguity

The degree of ambiguity of the retinal interface is calculated using the automatic segmentation model described above. In the model, among the 512 points, which is the number of vertical pixels per A-scan, those corresponding to the boundary layer were initially calculated for each A-scan of the OCT image, where the probability distribution of the boundary layer was the output, and the point with the highest probability was detected as the boundary layer. If the probability distribution is not biased toward one point and varies, the detection of the boundary layer is considered uncertain. The variation in this probability distribution was calculated using entropy. Entropy is an index indicating the degree of chaos and irregularity of a state, and is calculated using the following formula:

The entropy value (Entropy_*x,l*_) at position *x* of boundary layer *l* of the A-scan is

$${Entropy}_{x,l}=-\sum_{z}p_{x,z,l}\;\log\;p_{x,z,l}$$

Here, *p_x,z,l_* is the output value of the network at the coordinates (*x,z*) of boundary layer *l*.

**Fig 2:**
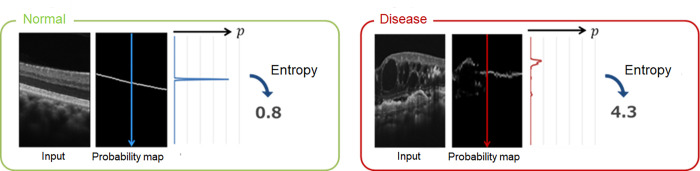
Conceptual diagram of the entropy value of each point in OCT A-scan. The OCT A-scan line in the normal eyes was blue, and in the disease-affected eyes, the scan line was red. In normal eyes, the probability distribution does not vary, so the entropy is small; however, in disease-affected eyes, the variation is large, so the entropy value is large.

**Fig 3:**
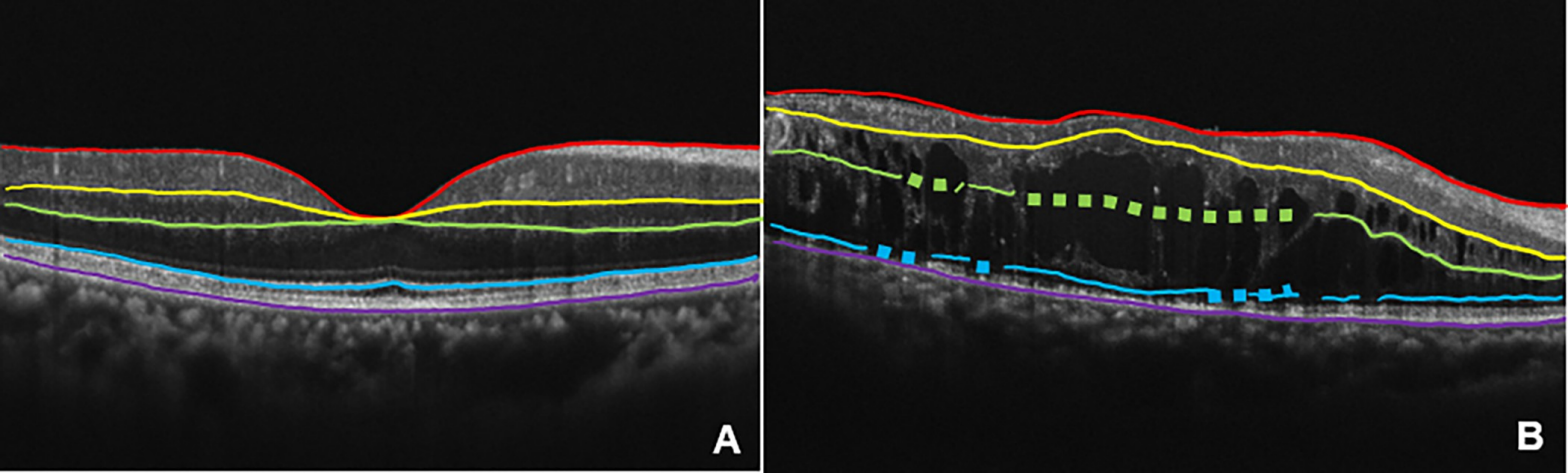
Example of segmentation using the ambiguity index. Normal eyes (A) and diabetic retinopathy (B). The boundary line of the layer was drawn using AI in this study. Because each boundary line is represented by a series of dots, there is an entropy value for each dot. The points where the value was high were delineated by a thick dotted line, and the points where the value was small were delineated by a thin solid line. Red; ILM; yellow, IPL/INL; green, OPL/ONL; blue, EZ; purple, RPE/BM.

In other words, the larger the entropy, the more scattered the probability distribution, and the more uncertain the layer detection. Entropy was calculated for each boundary layer of the retina in the OCT image, with the average value defined as the ambiguity index (Amb-I). The lower the Amb-I, the smaller the variation in the probability distribution; the higher the Amb-I, the greater the variation in the probability distribution (Fig 2). An example of segmentation using Amb-I is shown in Fig 3.

### Heatmap/Ambiguity map creation

**Fig 4:**
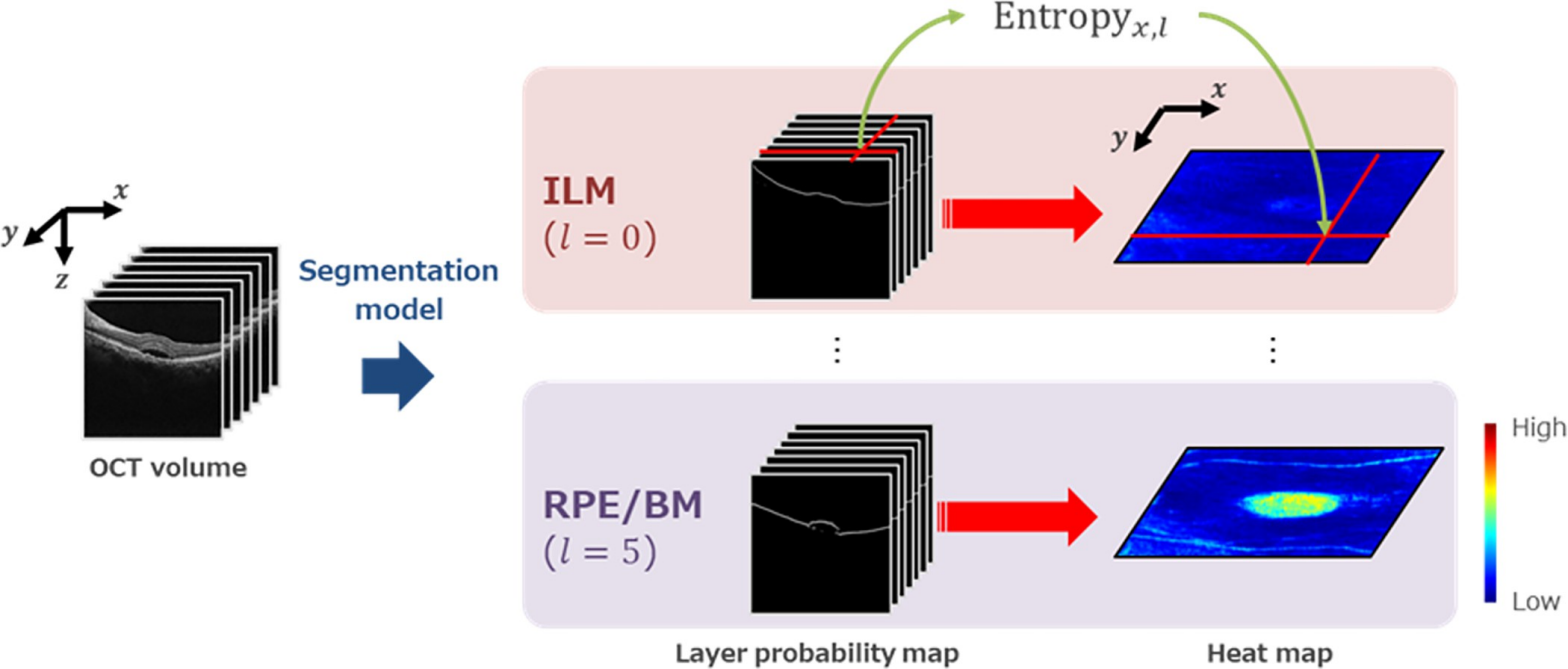
Scheme of ambiguity map productive process. By calculating entropy in the volume scan, it is possible to create a heatmap for each layer of the retina and visualize the part with high entropy.

In the proposed method, a heat map for each layer can be created by arranging the entropy calculated for each A-scan in the OCT volume (Fig 4**).** The created heatmap was smoothened using a Gaussian filter with σ = 1 and normalized such that the minimum value would be 0 and the maximum value would be 1. A jet color map was applied to color the normalized heat map, the result of which is called an ambiguity map.

### Image labeling

Two retinal experts labeled the presence or absence of abnormalities in each layer of the OCT image. Those with an epiretinal membrane (ERM), retinal edema, hard exudate, retinal pigment epithelium (RPE) abnormality, serous retinal detachment (SRD), pigment epithelial detachment (PED), and drusen were defined as abnormalities. The retina was also examined to determine the layer in which these abnormalities were observed. If two examiners had different opinions, abnormalities were established after discussions between the two examiners.

### Statistical analysis

All statistical analyses were performed using SPSS Statistics 19 for Windows (SPSS Inc., IBM, Somers, NY, USA). The difference between the mean values of normal and abnormal Amb-I levels was examined using the Mann-Whitney U test. The ability to classify normal and abnormal parts was evaluated using the AUC of the ROC curve. A p-value of 0.05 or less was considered significant.

## Results

After consultation with two experts (HS and SS), if the diagnosis was split, a third expert (HT) was included, and the images were classified into 189 normal and 111 disease-affected eyes. Thirty patients had abnormalities at the boundary between the internal limiting membrane (ILM), 44 at the boundary between the nerve fiber layer (NFL) and ganglion cell layer (GCL), 50 at the boundary between the inner plexiform layer (IPL) and inner nuclear layer (INL), 48 at the boundary between the outer plexiform layer (OPL) and outer nuclear layer (ONL), 78 in the ellipsoid zone (EZ), and 48 at the boundary between the retinal pigment epithelium (RPE) and Bruch’s membrane (BM).

### Relationship between the presence or absence of disease and overall ambiguity

**Fig 5:**
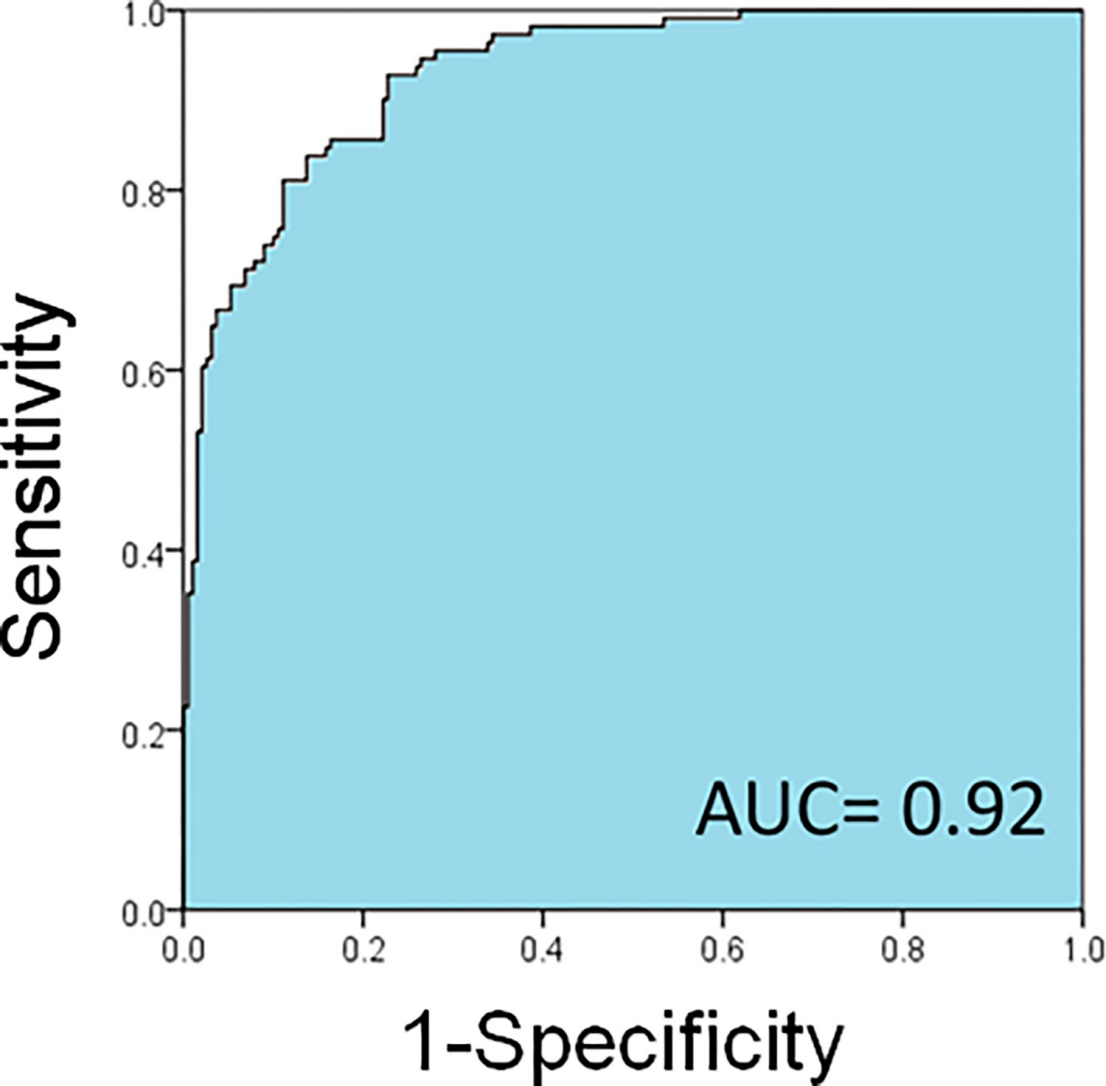
The ROC curve for the classification of normal and abnormal images by mean Amb-I of each layer of the retina, classified by AUC = 0.92.

The overall Amb-I of the normal image was 1.76 ± 0.10, and the Amb-I of the diseased image was 2.06 ± 0.22, showing a significant difference (p < 0.05, Mann-Whitney U test) (Table 1). Fig 5 shows the ROC curve for the classification of normal and diseased images using the Amb-I. The AUC for this ROC curve was 0.92. We suggest that it should be possible to classify diseased and normal eyes using Amb-I.

**Table 1:** Ambiguity index of each layer in normal and diseased eyes.

	Amb-I of normal eyes (n = 189)	Amb-I of diseased eye (n = 111)	p-value
**All**	1.76 ± 0.10	2.06 ± 0.22	< 0.001
**ILM**	1.67 ± 0.12	1.70 ± 0.14	0.113
**NFL/GCL**	1.91 ± 0.20	2.32 ± 0.29	< 0.001
**IPL/INL**	2.12 ± 0.23	2.64 ± 0.31	< 0.001
**OPL/ONL**	1.96 ± 0.29	2.42 ± 0.27	< 0.001
**EZ**	1.55 ± 0.22	2.12 ± 0.43	< 0.001
**RPE/BM**	1.58 ± 0.18	1.93 ± 0.32	< 0.001

Amb-I: ambiguity index

### Relationship between the boundary abnormality of each layer and Amb-I

**Fig 6:**
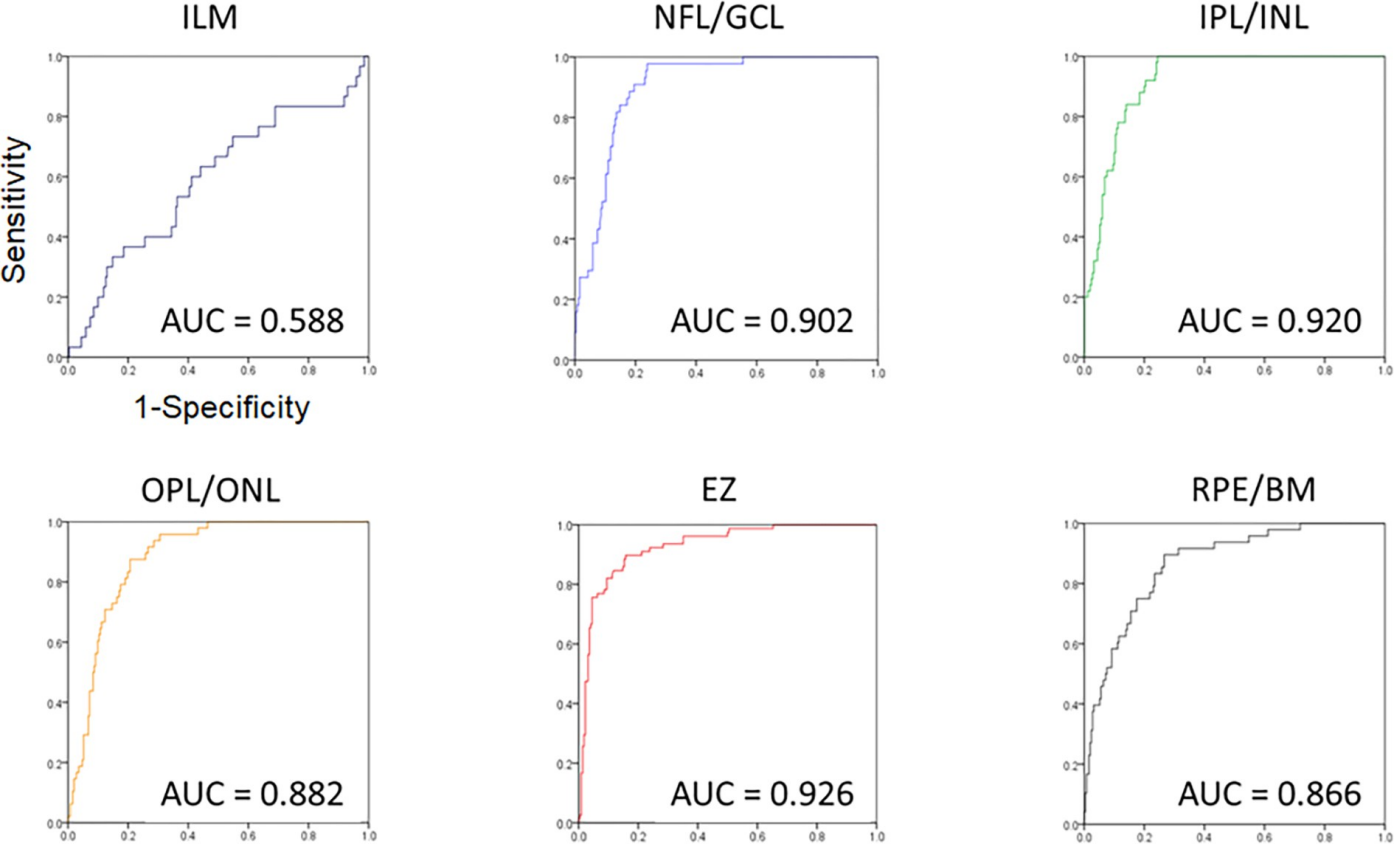
Ambiguity index distribution map. Blue: Normal Amb-I distribution map; Red: Abnormal Amb-I distribution map. It was observed that IPL/INL and EZ can be classified with high accuracy.

Fig 6 shows the ROC curve of the image classification for each layer of the retina. The Amb-I ROC curve for the presence or absence of ILM boundary abnormalities had AUC = 0.588. The Amb-I ROC curve for the presence or absence of NFL/GCL boundary abnormalities had an AUC of 0.902. The Amb-I ROC curve for the presence or absence of IPL/INL boundary abnormalities had AUC of 0.920. The Amb-I ROC curve for the presence or absence of OPL/ONL boundary abnormalities had AUC of 0.882. The Amb-I ROC curve for the presence or absence of EZ had AUC of 0.926. The Amb-I ROC curve for the presence or absence of RPE/BM boundary abnormalities had AUC = 0.866. Representative cases are shown in S2 Fig.

### Ambiguity map

A Heatmap/Ambiguity-Map was created, and representative cases were presented to reveal the usefulness of this algorithm.

**Fig 7:**
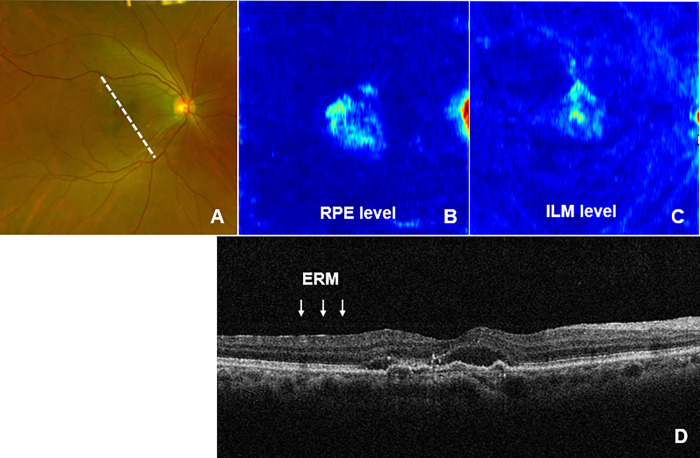
Ocular findings of case 1. (A) Color fundus photograph. (B) Amb-I heatmap image at RPE level. (C) Amb-I heatmap image at ILM level. (D) OCT B-scan image, scan of the white part of the dashed line in (A). Amb-I: ambiguity index, RPE: retinal pigment epithelium, ILM: internal limiting membrane, ERM: epiretinal membrane.

Case 1: A 78-year-old male was treated for neovascular AMD for three years. Routine examination of the ocular fundus with OCT and color photography revealed that the physicians did not notice the ILM layer lesion. The Ambiguity-Map image revealed the presence of a high Amb-I spot on the ILM layer in addition to the pigment epithelial layer. After careful examination of the OCT B-scan image, an epiretinal membrane was found (Fig 7). The epiretinal membrane was not noticed until the Ambiguity-Map. This case shows that the Ambiguity-Map can reveal some important lesions that are often overlooked in cases of other evident diseases. Although this ERM does not require treatment at this time, many ERMs worsen over time and require careful observation in the future. It is thanks to this AI that we were able to identify such findings early. Early detection will ensure a better decision on the appropriate time for treatment, and this thought process can be described as "wayfinding".

Case 2: A 54-year-old male with simple diabetic retinopathy.

**Fig 8:**
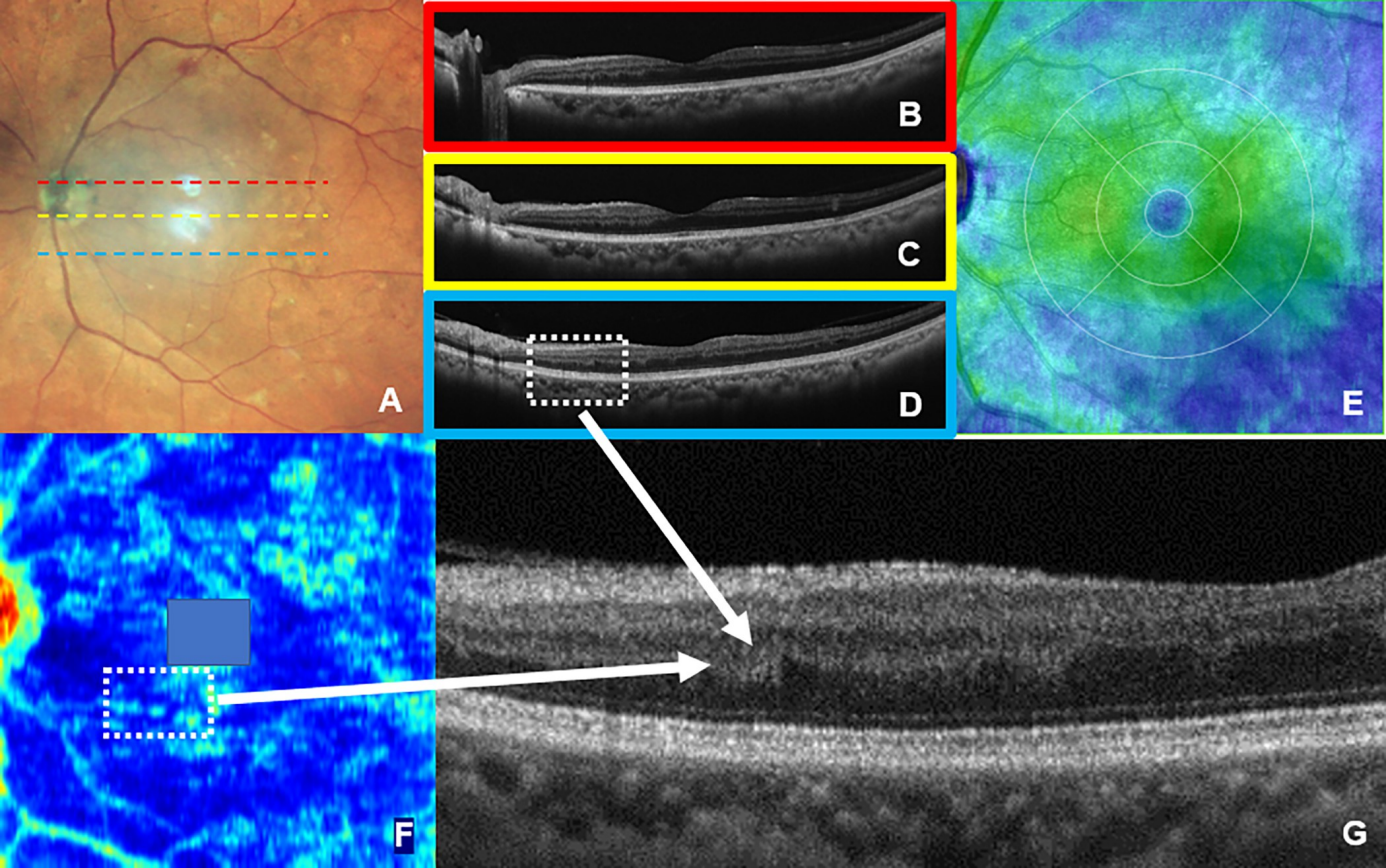
Ocular findings of case 2. Color fundus photography (A). OCT image of A-scan with dotted red, yellow, and blue lines (B, C, and D). The color map (E) reflects the thickness of the retina in the same case. These findings are consistent with those of simple diabetic retinopathy. However, when the heatmap (F) based on Amb-I shows an image of the OPL/ONL boundary layer, a light-blue island with a large Amb-I is observed in the layer below the macula. Therefore, if that part is magnified (dotted line qualification part of D and F), the presence of a distortion in the OPL/ONL (G) can be observed. From this, it can be determined that distortion begins at the layer structure of the retina, despite simple retinopathy.

The color fundus photograph, B-scan OCT image, and thickness map supported the diagnosis of simple diabetic retinopathy. The Ambiguity map of the OPL/ONL boundary layer shows some light blue islands. Then, looking at the indicated lesion carefully at high magnification, a distortion in the OPL/ONL could be found. This case shows that subtle but important lesions can be found in the Ambiguity map (Fig 8).

**Fig 9:**
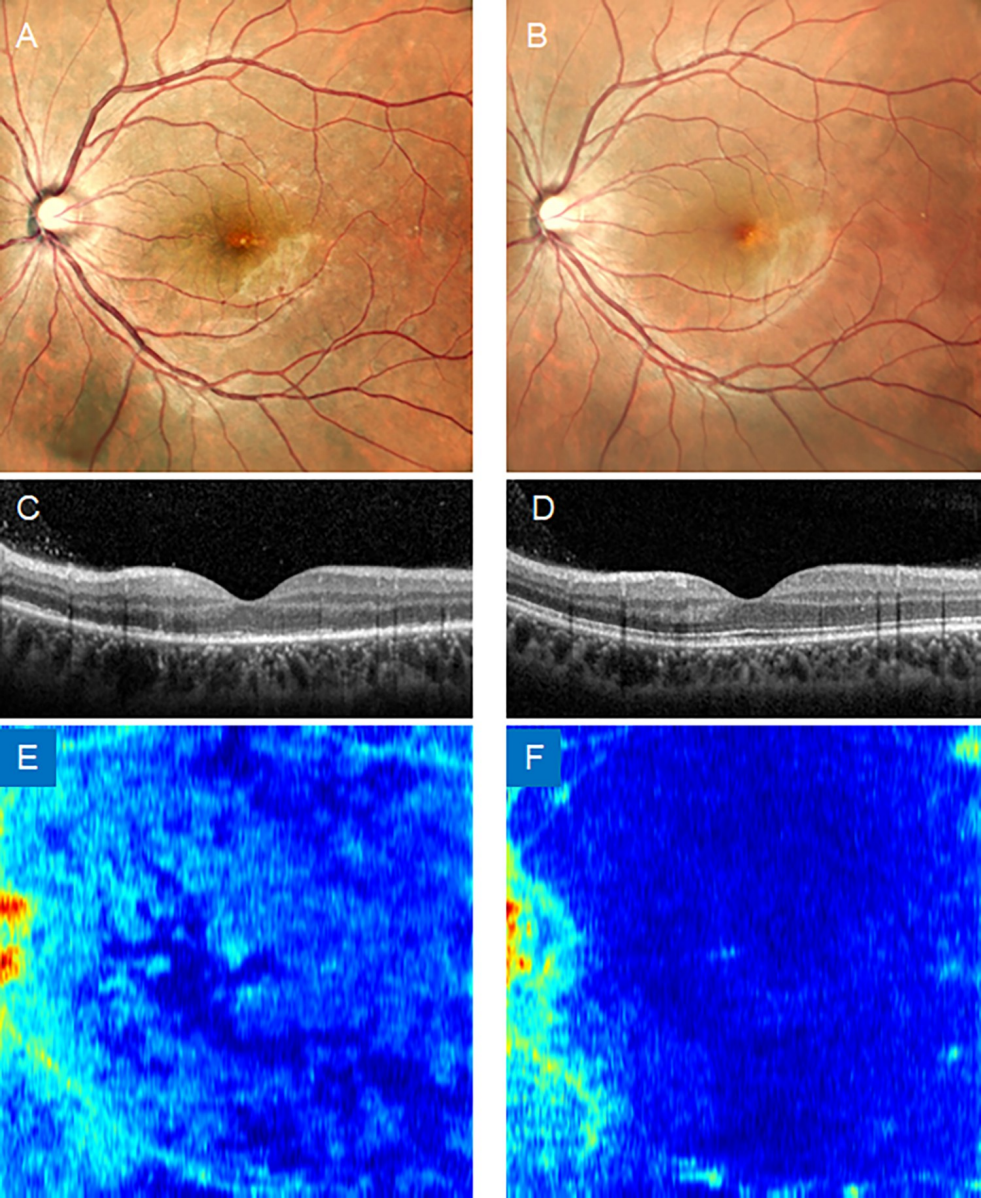
Ocular findings of case 3. Color fundus photographs at the first visit (A) and after 3 months (B). B-scan OCT image at the first visit (C) and after 3 months (D). EZ level Amb-I heatmap image at the first visit (E) and after three months (F). Amb-I: ambiguity index; EZ: ellipsoid zone.

Case 3: The patient was a 31-year-old female with acute zonal occult outer retinopathy (AZOOR). At the initial visit, although fundus photography showed no apparent changes, OCT revealed that the normal outer retinal structure was impaired, which was consistent with AZOOR. The area of the impaired retina can be clearly seen as light blue-colored areas in the Ambiguity map image. After three months, the impaired lesions disappeared on fundus photography and OCT. This was confirmed using an Ambiguity map image. This case showed that the chronological change in the lesion became evident in the Ambiguity-Map image (Fig 9).

## Discussion

The existing automatic segmentation model of OCT B-scan images using deep learning enables the quantification of the ambiguity of the boundary surface determination of each layer of the retina. By applying quantified data, it is possible to detect abnormal or ambiguous parts with a specific value, Amb-I. Above all, the Ambiguity-Map enabled the detection of abnormal areas instantly and correctly.

There have been reports of the use of deep learning to classify OCT normals and abnormalities with high accuracy [22, 23]. However, deep learning has a problem that the analysis process is a black box and it is difficult to clarify the reason for the classification [12] =. This is a major obstacle in the clinical application of AI. In this study, abnormalities were detected by quantifying the analysis process and it was possible to numerically determine why the abnormalities were classified as they were.

The advantage of this method is that it is easy to understand the degree of abnormality, because quantified numerical values can be obtained. In addition, because abnormalities can be detected for each layer, it is easy to determine which layer has an abnormality. The indicated areas could then be re-examined carefully using OCT. We believe that repeating the processes of physicians concerning these parts can facilitate correct diagnoses rather than providing the final diagnosis. This is consistent with the concept of wayfinding. Moreover, as the segmentation model was applied, it was not necessary to incorporate a new program for abnormality detection into the machine. Previously, an OCT segmentation model was applied to an automatic diagnostic model [24]. However, in this case, transfer learning using a deep learning model was performed in the diagnostic process, and because the diagnostic process is a black box, it is very different from this model. Seeboeck et al. used uncertainty to detect anomalies in OCT [17]. In their study, uncertainty was used to infer dropouts in order to realize a neural network, and because no explanation could be given as to why the dropout occurred, it is difficult to conclude that their algorithm was an explainable AI. Another difference is the high cost of computational processing compared with that described in the present study, where anomalies were detected using simple entropy. Yim et al. recently developed an AI algorithm for OCT images of suspected AMD that points to the location of findings such as pigment epithelial detachment, which are important for AMD diagnosis [25]. Although this is a breakthrough, it remains unclear how well it can be adapted t like o diseased eyes other than AMD. By contrast, our AI is applicable to all retinal diseases. From a liability perspective, this AI, which points out lesions, is useful because the final diagnosis is still and will always be made by humans. Again, the algorithm developed by Yim et al. requires a large GPU and thus a large computation time, as found in a study by Seebock et al. Our algorithm is simple and can be implemented using current OCT instruments with a short computation time.

The ability to detect abnormalities at the ILM interface was not high, according to the AUC obtained in terms of specificity and sensitivity. The lesions on the ILM interface were mainly ERM, but in the OCT image of ERM, the brightness of only the lesion site increased. This was probably because the entropy value often remained low in the segmentation, such that there were many cases where the boundary surface was not disturbed. Furthermore, the inclusion of cases with a narrow range of ERM is also considered to be one of the factors. In contrast, strong abnormalities were detected between the IPL/INL interface and the EZ. Diabetic macular edema is a typical disease that causes IPL/INL abnormalities, while AMD and central serous chorioretinopathy (CSC) are typical diseases that cause EZ abnormalities. However, it is probable that the entropy value was high and the detection power was high because of the significant changes in the OCT image caused by the lesion.

In this study, the entropy of each layer of OCT B-scan images in one cross-section passing through the macula was averaged and calculated as Amb-I. Note that by taking the average, small changes that should be detectable may be missed. However, by applying this method to the OCT volume scan and displaying the entropy of each measurement point on a color scale and applying a mapping, the lesion site can be visually comprehended, as shown in Figs 7–9. Distortions in the retinal layer can also be visualized in cases of early diabetic retinopathy (Fig 8). It can be argued that a change in this magnitude is not a type of wayfinding because it does not affect the treatment. However, our definition of “wayfinding” includes not only findings that immediately affect treatment, but also subtle abnormal findings that may affect treatment in the future. It is therefore important to not miss such subtle findings in diabetic retinopathy.

To identify unexpected lesions that are often overlooked in regular medical care, for example, when treating age-related macular degeneration, the doctor may concentrate on the macula while often overlooking mild ERM; however, as shown in Fig 7, ERM can be diagnosed at an early stage. In this method, an area around the abnormal area is attached and scanned to obtain an OCT B-scan image. With this software, the average entropy value of each layer was calculated, and it was possible to numerically indicate the layer with an abnormality. In other words, in the heatmap, the area where there is a high possibility of any abnormality is marked and scanned with OCT, making it possible to indicate the cross section of the image that is abnormal. Most importantly, what makes this method useful is that ambiguous areas can be seen at a glance. Numerical results are useful; however, in busy outpatient settings, abnormal areas may be overlooked. The heatmap method, which shows the location of the lesion with the intensity of ambiguity, has a great advantage in busy outpatient clinics, because this information is instantly available. However, owing to the absence of the inner retinal layer, it should be noted that Amb-I is higher in the central fovea. Care should therefore be taken and processing should be implemented from the beginning.

In clinical practice, it is necessary to identify abnormal areas within a short timeframe. Because numerous OCT image slices per case need to be carefully observed within a short timeframe, it is difficult to find new lesions (particularly small or subtle ones) that appear in areas outside the main lesion. This occurs because humans tend to look carefully at slices that contain major lesions but not as carefully at slices that do not. However, such small and often-overlooked lesions may be precursors to major changes in the future. Therefore, by creating a heat map/ambiguity map, we made it possible to easily observe the overall areas. This provides a rough distribution of lesions within a short timeframe and is an extremely useful tool for clinicians. In our experience, other than the main lesion, this has significantly reduced the number of abnormal areas missed.

It should be noted that this model is unsuitable for the final diagnosis of a specific disease. Although many AI systems provide a final diagnosis, this is not the intent of our developed AI, which is not considered a major drawback. This AI pinpoints “abnormal” images in an OCT image that are otherwise invisible in a fundus image or through a single-section OCT. The AI used for diagnosis is trained on annotated data for each disease, and thus it can make diagnoses that follow the annotations but cannot make diagnoses for other diseases. However, in clinical practice, multiple lesions, borderline lesions, and small early lesions that cannot be diagnosed are hidden, and many diseases that cannot be annotated are important. We believe that our method can compensate for the shortcomings of existing models.

Should a person specifically identify an overlooked or unnoticed abnormality, they would consider it and determine the next necessary test for the examination, which would finally lead to a correct diagnosis. Wayfinding AI is yet to be clearly defined. However, the AI developed in this study is in line with the idea of Wayfinding AI, in that reaching the correct diagnosis through trial and error helps human thinking, and this diagnosis process has been adopted for a long time. The proposed AI is a new AI that would be more acceptable to doctors than normal AI, which remains a black box in the diagnostic process. Overdependence on AI may result in unthinkable misdiagnoses, which can be prevented by the proposed AI.

## Supporting information

S1 Fig The layer on the tissue and the boundary on the OCT image. ILM: internal limiting membrane (ILM), NFL: nerve fiber layer, GCL: ganglion cell layer, IPL: inner plexiform layer, INL: inner nuclear layer, OPL: outer plexiform layer, ONL: outer nuclear layer, EZ: ellipsoid zone, RPE: retinal pigment epithelium, BM: Bruch’s membrane.
(PNG)

S2 Fig OCT B-scan image of eye diseases and ambiguity index of each layer. (A) Normal, (B) diabetic macular edema, and (C) age-related macular degeneration. The Amb-I of each layer of the retina showed a relatively low value in the normal eye. However, diseases cause abnormalities in each layer of the retina, such as diabetic macular edema, which displays a high Amb-I value in all layers except ILM. In contrast, EZ shows a relatively high value in diseases that cause abnormalities only in the outer layer of the retina (i.e., atrophic AMD), whereas it is relatively low in the inner layer of the retina.
(PNG)

## References

[1] LeCun Y, Bengio Y, Hinton G. Deep learning. Nature. 2015;521(7553): 436–444. doi: 10.1038/nature14539 26017442

[2] Gulshan V, Peng L, Coram M, Stumpe MC, Wu D, Narayanaswamy A, et al. Development and validation of a deep learning algorithm for detection of diabetic retinopathy in retinal fundus photographs. JAMA. 2016;316(22): 2402–2410. doi: 10.1001/jama.2016.17216 27898976

[3] Abràmoff MD, Lou Y, Erginay A, Clarida W, Amelon R, Folk JC, et al. Improved automated detection of diabetic retinopathy on a publicly available dataset through integration of deep learning. Invest Ophthalmol Vis Sci. 2016;57(13): 5200–5206. doi: 10.1167/iovs.16-19964 27701631

[4] Gargeya R, Leng T. Automated identification of diabetic retinopathy using deep learning. Ophthalmology. 2017;124(7): 962–969. doi: 10.1016/j.ophtha.2017.02.008 28359545

[5] Ting DSW, Cheung CY-L, Lim G, Tan GSW, Quang ND, Gan A, et al. Development and validation of a deep learning system for diabetic retinopathy and related eye diseases using retinal images from multiethnic populations with diabetes. JAMA. 2017;318(22): 2211–2223. doi: 10.1001/jama.2017.18152 29234807 PMC5820739

[6] Burlina PM, Joshi N, Pekala M, Pacheco KD, Freund DE, Bressler NM. Automated grading of age-related macular degeneration from color fundus images using deep convolutional neural networks. JAMA Ophthalmol. 2017;135(11): 1170–1176. doi: 10.1001/jamaophthalmol.2017.3782 28973096 PMC5710387

[7] Burlina PM, Joshi N, Pacheco KD, Freund DE, Kong J, Bressler NM. Use of deep learning for detailed severity characterization and estimation of 5-year risk among patients with age-related macular degeneration. JAMA Ophthalmol. 2018;136(12): 1359–1366. doi: 10.1001/jamaophthalmol.2018.4118 30242349 PMC6583826

[8] Li Z, He Y, Keel S, Meng W, Chang RT, He M. Efficacy of a deep learning system for detecting glaucomatous optic neuropathy based on color fundus photographs. Ophthalmology. 2018;125(8): 1199–1206. doi: 10.1016/j.ophtha.2018.01.023 29506863

[9] Schmidt-Erfurth U, Sadeghipour A, Gerendas BS, Waldstein SM, Bogunović H. Artificial intelligence in retina. Prog Retin Eye Res. 2018;67: 1–29. doi: 10.1016/j.preteyeres.2018.07.004 30076935

[10] Grassmann F, Mengelkamp J, Brandl C, Harsch S, Zimmermann ME, Linkohr B, et al. A deep learning algorithm for prediction of age-related eye disease study severity scale for age-related macular degeneration from color fundus photography. Ophthalmology. 2018;125(9): 1410–1420. doi: 10.1016/j.ophtha.2018.02.037 29653860

[11] Brown JM, Campbell JP, Beers A, Chang K, Ostmo S, Chan RVP, et al. Automated diagnosis of plus disease in retinopathy of prematurity using deep convolutional neural networks. JAMA Ophthalmol. 2018;136(7): 803–810. doi: 10.1001/jamaophthalmol.2018.1934 29801159 PMC6136045

[12] Castelvecchi D. Can we open the black box of AI? Nature. 2016;538(7623): 20–23. doi: 10.1038/538020a 27708329

[13] Keel S, Wu J, Lee PY, Scheetz J, He M. Visualizing deep learning models for the detection of referable diabetic retinopathy and glaucoma. JAMA Ophthalmol. 2019;137(3): 288–292. doi: 10.1001/jamaophthalmol.2018.6035 30570648 PMC6440231

[14] Ting DSW, Pasquale LR, Peng L, Campbell JP, Lee AY, Raman R, et al. Artificial intelligence and deep learning in ophthalmology. Br J Ophthalmol. 2019;103(2): 167–175. doi: 10.1136/bjophthalmol-2018-313173 30361278 PMC6362807

[15] Adler-Milstein J, Chen JH, Dhaliwal G. Next-Generation Artificial Intelligence for Diagnosis: From Predicting Diagnostic Labels to “Wayfinding”. JAMA. 2021;326(24): 2467–2468. doi: 10.1001/jama.2021.22396 34882190 PMC12049696

[16] Fujimoto J, Swanson E. The Development, Commercialization, and Impact of Optical Coherence Tomography. Invest Ophthalmol Vis Sci. 2016; 57(9):OCT1–OCT13. doi: 10.1167/iovs.16-19963 27409459 PMC4968928

[17] Seeböck P, Orlando JI, Schlegl T, Waldstein SM, Bogunović H, Klimscha S, et al. Exploiting epistemic uncertainty of anatomy segmentation for anomaly detection in retinal OCT. IEEE Trans Med Imaging. 2020;39(1): 87–98. doi: 10.1109/TMI.2019.2919951 31170065

[18] Sonoda S, Shiihara H, Terasaki H, Kakiuchi N, Funatsu R, Tomita M, et al. Artificial intelligence for classifying uncertain images by humans in determining choroidal vascular running pattern and comparisons with automated classification between artificial intelligence. PLOS ONE. 2021;16(5): e0251553. doi: 10.1371/journal.pone.0251553 33989334 PMC8121314

[19] Hussain S, Guo F, Li W, Shen Z. DilUnet: A U-net based architecture for blood vessels segmentation. Comput Methods Programs Biomed. 2022;218:106732. doi: 10.1016/j.cmpb.2022.106732 35279601

[20] Ronneberger O, Fischer P, Brox T, editors. U-net: Convolutional networks for biomedical image segmentation. International Conference on Medical image computing and computer-assisted intervention. Springer, Cham. 2015;234–231. doi: 10.1007/978-3-319-24574-4_28

[21] He Y, Carass A, Liu Y, Jedynak BM, Solomon SD, Saidha S, et al. Fully Convolutional Boundary Regression for Retina OCT Segmentation. Med Image Comput Comput Assist Interv. 2019;11764:120–128. doi: 10.1007/978-3-030-32239-7_14 31853524 PMC6918831

[22] Lee CS, Baughman DM, Lee AY. Deep learning is effective for classifying normal versus age-related macular degeneration OCT images. Ophthalmol Retina. 2017;1(4): 322–327. doi: 10.1016/j.oret.2016.12.009 30693348 PMC6347658

[23] De Fauw J, Ledsam JR, Romera-Paredes B, Nikolov S, Tomasev N, Blackwell S, et al. Clinically applicable deep learning for diagnosis and referral in retinal disease. Nat Med. 2018;24(9): 1342–1350. doi: 10.1038/s41591-018-0107-6 30104768

[24] Rim TH, Lee AY, Ting DS, Teo KYC, Yang HS, Hyeonmin K, et al. Computer-aided detection and abnormality score for the outer retinal layer in optical coherence tomography. Br J Ophthalmol. 2021;106(9):1301–1307. doi: 10.1136/bjophthalmol-2020-317817 33875452 PMC9208332

[25] Yim J, Chopra R, Spitz T、Winkens J, Obika A, Kelly C, et al. Predicting conversion to wet age-related macular degeneration using deep learning. Nat Med. 2020;26: 892–899. doi: 10.1038/s41591-020-0867-7 32424211

