# Supplementary figures and images for: Wayfinding artificial intelligence to detect clinically meaningful spots of retinal diseases: Artificial intelligence to help retina specialists in real world practice
Source: PLoS One. 2023 Mar 27;18(3):e0283214. doi: 10.1371/journal.pone.0283214 (PMC10042340; doi:10.1371/journal.pone.0283214)

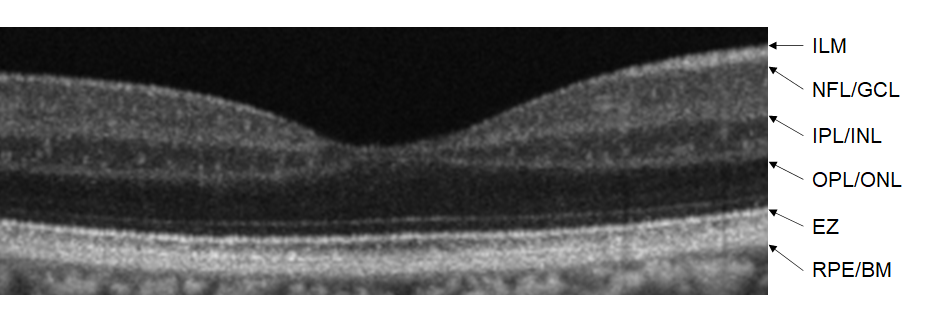

Supplement: S1 Fig — ILM: internal limiting membrane (ILM), NFL: nerve fiber layer, GCL: ganglion cell layer, IPL: inner plexiform layer, INL: inner nuclear layer, OPL: outer plexiform layer, ONL: outer nuclear layer, EZ: ellipsoid zone, RPE: retinal pigment epithelium, BM: Bruch’s membrane. (PNG) [file pone.0283214.s001.png]

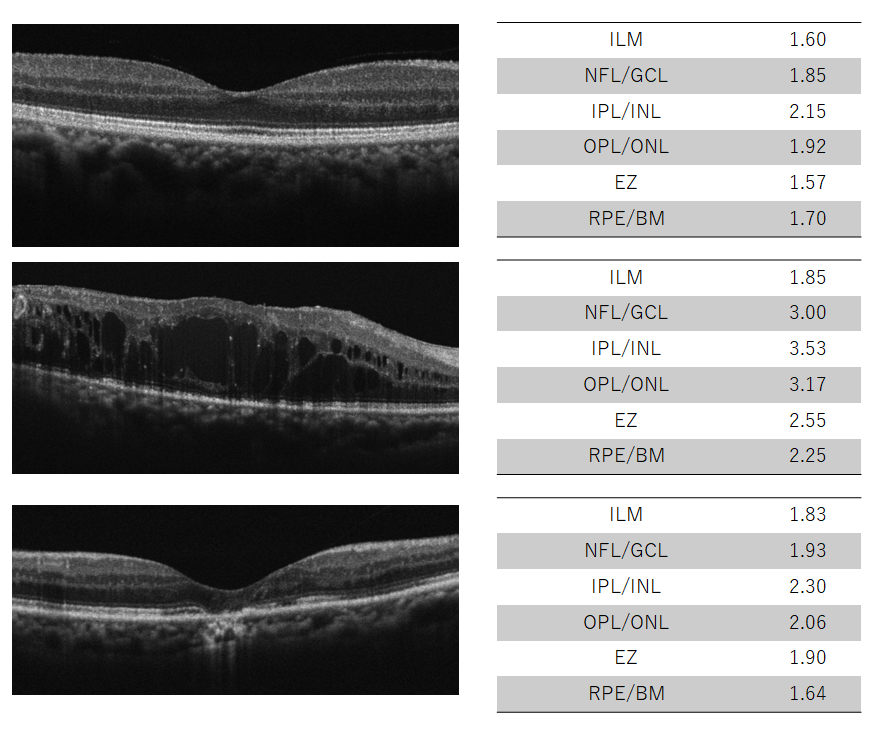

Supplement: S2 Fig — (A) Normal, (B) diabetic macular edema, and (C) age-related macular degeneration. The Amb-I of each layer of the retina showed a relatively low value in the normal eye. However, diseases cause abnormalities in each layer of the retina, such as diabetic macular edema, which displays a high Amb-I value in all layers except ILM. In contrast, EZ shows a relatively high value in diseases that cause abnormalities only in the outer layer of the retina (i.e., atrophic AMD), whereas it is relatively low in the inner layer of the retina. (PNG) [file pone.0283214.s002.png]
